# Spontaneous Spinal Epidural Hematoma During Pregnancy: A Case Report

**DOI:** 10.7759/cureus.72971

**Published:** 2024-11-04

**Authors:** Merih C Yilmaz, Keramettin Aydin

**Affiliations:** 1 Neurosurgery, VM Medical Park Samsun Hospital, Samsun, TUR

**Keywords:** laminectomy, myelomalacia, paraplegia, pregnancy, spontaneous spinal epidural hematoma

## Abstract

Spontaneous spinal epidural hematoma (SSEH) during pregnancy is an extremely rare neurological emergency, often presenting with sudden neck pain and progressive limb weakness. This report describes a 26-year-old pregnant woman at 26 weeks of gestation who developed paraplegia and severe muscle weakness without prior trauma. MRI revealed a cervical epidural hematoma, leading to an emergency C6 and C7 laminectomy. Despite surgery, the patient remained quadriplegic, with follow-up MRI showing persistent myelomalacia. This case highlights the challenges of diagnosing and managing SSEH during pregnancy, emphasizing the need for prompt intervention and ongoing research to improve outcomes for affected patients.

## Introduction

Pregnancy is a critical and delicate phase in a woman's life. However, unforeseen events during this process can be alarming for both expectant mothers and healthcare providers. One such condition is the spontaneous occurrence of cervical epidural hematoma during pregnancy. A cervical epidural hematoma typically occurs without any injury or apparent cause, presenting as a sudden-onset condition that can have severe consequences. Recent case reports and studies have indicated that both COVID-19 vaccination and infection may play a role in the etiology of spontaneous epidural hematomas [1,2].

This condition, which is rare and occurs spontaneously during pregnancy, is often a result of bleeding in the cervical region [3]. Epidural hematomas, caused by the accumulation of blood in the epidural space outside the spinal cord, can lead to severe complications. This situation poses a risk to the mother's life and the baby's health. In this article, we will explore a case presentation to understand spontaneous spinal epidural hematoma (SSEH) cases and guide individuals and healthcare professionals on how to respond when faced with this condition.

It is essential to have access to precise information and effective treatment methods when dealing with rare but potentially serious medical conditions like cervical epidural hematoma during pregnancy as insufficient knowledge can lead to inaccurate and ineffective treatment of patients [4]. Conservative and decompressive surgical options are offered based on the findings from radiological and neurological evaluations. This article will provide a detailed analysis of the clinical characteristics, diagnostic techniques, and treatment alternatives for spontaneous cervical epidural hematomas occurring during pregnancy. The goal is to bridge the knowledge gap in this field and to assist individuals in understanding and managing this condition, which may progress despite opportunities for early intervention. Additionally, it seeks to provide informative guidance for healthcare professionals dealing with such cases.

## Case presentation

**Figure 1:**
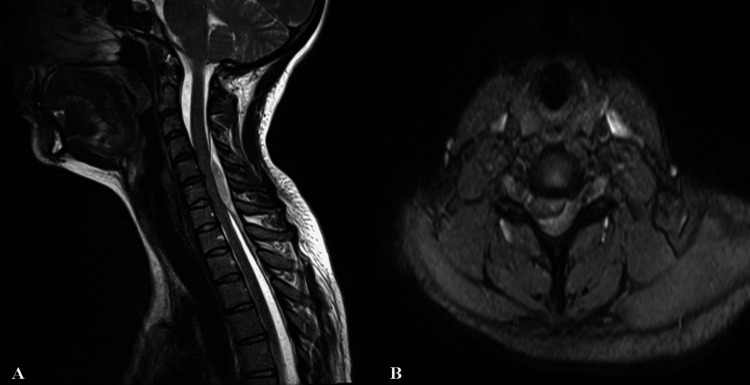
Preoperative cervical sagittal (A) and axial (B) MR images of the patient revealing an epidural hematoma exerting a compressive effect from the right posterolateral aspect

A 26-year-old pregnant woman, at 26 weeks of gestation, presented to the emergency department with a two-day history of neck pain and progressively worsening weakness in all four limbs. She had no previous history of trauma, no notable medical history, was not taking any medications, and had no smoking history. Patient's platelet level, prothrombin time, activated partial thromboplastin time and fibrinogen level were within the normal range. A neurological examination revealed paraplegia and severe muscle weakness in the bilateral upper extremities, graded as 1/5 (American Spinal Injury Association [ASIA] Impairment Scale, ASIA C). The patient noted that these symptoms had emerged on the same day. Cranial and cervical MRI scans were conducted, identifying an epidural hematoma in the cervical region (Figure 1).

**Figure 2:**
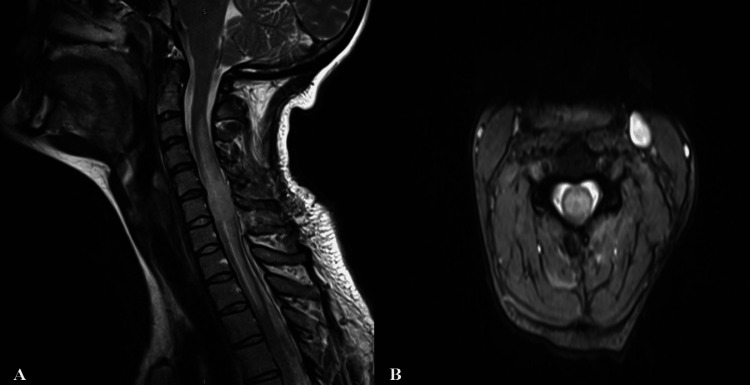
Cervical sagittal (A) and axial (B) MR images of the patient on the seventh postoperative day demonstrating diffuse myelomalacia

Emergency surgery was advised, and the patient was thoroughly informed about the procedure before obtaining consent. Following a C6 and C7 cervical laminectomy, the epidural hematoma was evacuated. Postoperatively, the patient was closely monitored in the intensive care unit. A follow-up cervical MRI conducted on the seventh postoperative day showed significant spinal cord edema at the C6-C7 levels (Figure 2).

Despite medical treatment, the patient remained quadriplegic upon discharge. Two years later, she continued to experience hypoesthesia below the T4 level, urinary incontinence, and muscle weakness in the bilateral upper extremities, graded as 4/5. She remained paraplegic (0/5) with no anal reflex. A cervical MRI performed two years later revealed myelomalacia at the C6 level. The baby was born healthy at 38 weeks, and the patient’s clinical monitoring and rehabilitation are currently in progress.

## Discussion

Spontaneous spinal epidural hematoma is an uncommon yet significant neurological emergency, representing 0.3% to 0.9% of all lesions occupying the epidural space [5]. Spontaneous epidural hematomas of the spine are rarely reported during pregnancy. Given that blood clotting tends to increase in pregnant women, the occurrence of bleeding disorders such as hematomas is generally not anticipated. Due to its infrequency and unusual symptoms, rapid diagnosis can be challenging, and the underlying causes are still not well understood [3].

The exact etiology of SSEH remains largely unknown. However, several predisposing factors have been identified that may contribute to its development. These include the use of anticoagulant medications, the presence of vascular malformations, underlying conditions such as hemophilia, and abuse of substances like cocaine [6-9]. Understanding these risk factors can help in identifying individuals at higher risk and in guiding preventive and therapeutic strategies.

In the majority of SSEH cases, the clinical presentation is characterized by acute and intense back or neck pain. This pain may start suddenly and can sometimes be triggered by minor activities such as defecation, lifting, coughing, or sneezing. However, more frequently, the pain begins spontaneously without any identifiable precipitating factors. Accompanying symptoms often include rapid development of spinal cord and nerve root dysfunction, which can swiftly progress to more severe manifestations such as paraparesis or tetraparesis [10].

Progressive neurological deficits in cases of SSEH are closely associated with the level of spinal involvement and the extent of nerve compression. The degree of neurological impairment often correlates with the severity of spinal cord or nerve root compression. Magnetic resonance imaging is the preferred diagnostic modality for evaluating SSEH, as it provides detailed visualization of the epidural space and the extent of hematoma. Due to the potential for false positives with computed tomography, MRI is preferred as the primary diagnostic tool. However, small arteriovenous malformations might not be detected by MRI. In cases where the clinical scenario justifies it, MR angiography and digital subtraction angiography of the spine can be utilized to improve the identification of such subtle vascular anomalies. SSEH most frequently occurs in the thoracic region of the spine. This predilection is attributed to the prominence of the epidural venous plexus in the thoracic spine, which is more developed compared to other spinal regions [11].

The prevailing hypothesis regarding the pathogenesis of SSEH suggests that it results from the rupture of epidural vessels within the low-pressure epidural space. This rupture is thought to occur in response to sudden increases in intra-abdominal or intra-thoracic pressure [12]. Hemodynamic changes during pregnancy can elevate epidural venous pressure, leading to vessel rupture and subsequent bleeding [13]. It has also been proposed that structural vascular alterations may result from elevated levels of estrogen and progesterone, in addition to various anatomical factors [14,15].

SSEH is typically located posteriorly or posterolaterally relative to the thecal sac [5]. In instances of extradural hematoma during pregnancy, improvement in neurological function has been seen after surgical intervention [16]. Some patients with mild neurological symptoms have been successfully managed with nonoperative treatments [17]. However, surgical intervention is advised for patients who exhibit neurological symptoms [18].

## Conclusions

SSEH during pregnancy, although rare, represents a significant neurological emergency that requires prompt diagnosis and intervention. This case highlights the critical importance of recognizing the condition's symptoms, such as acute neck pain and progressive limb weakness, which can arise without prior trauma. Despite the challenges associated with early diagnosis due to its infrequent occurrence and atypical presentation, MRI remains the gold standard for accurate assessment.

In this case, despite emergency surgical intervention and postoperative care, the patient experienced persistent neurological deficits. This outcome emphasizes the need for a comprehensive approach to treatment and rehabilitation. Future research should focus on better understanding of the pathophysiological mechanisms underlying SSEH, particularly in the context of pregnancy-related hemodynamic changes and hormonal influences. Additionally, further studies are warranted to refine treatment protocols and improve outcomes for affected patients. Effective management of SSEH requires a multidisciplinary approach, involving neurosurgeons, obstetricians, and rehabilitation specialists, to optimize both immediate and long-term care.
